# Assessment of Patient Use of a New Approach to Access Health Record Data Among 12 US Health Systems

**DOI:** 10.1001/jamanetworkopen.2019.9544

**Published:** 2019-08-14

**Authors:** Julia Adler-Milstein, Christopher Longhurst

**Affiliations:** 1 School of Medicine, University of California, San Francisco; 2 UC San Diego Health, San Diego, California

## Abstract

This cross-sectional study measures patient use of smartphone applications to download health record data.

## Introduction

Recent policy efforts have enhanced patients’ access to their electronic health information with a foundational component that allows them to download health record data to their smartphones.^1^ To enable this, health systems first deploy a set of technical and security standards that offer patients access to their electronic health information; patients must then activate the connection between their health system’s records and their smartphones. Patients who activate this connection go beyond viewing their data on a patient portal by transmitting data elements, such as current medications, test results, and immunization history, to a smartphone, where they can control them. Ultimately, the vision is for the emergence of an ecosystem in which third-party applications help patients and their clinicians and other caregivers convert data into health-improvement actions. We sought to create national measures of patient uptake of this newly available ability to establish a baseline against which to measure future progress and ecosystem evolution.^2^

## Methods

For this cross-sectional study, we identified 12 geographically diverse US health systems with at least 9 months of experience (beginning in April 2018) allowing patients to download their electronic health information to a smartphone (via Fast Healthcare Interoperability Resources–enabled application programming interfaces [APIs]). All health systems used an electronic health record from the same vendor (Epic), which allowed us to create a standard data specification that the vendor executed on behalf of each participating health system. The data specification included monthly and cumulative counts of unique patient users as well as the number of unique patients who had logged into the patient portal each month, the latter of which served as a denominator for patients eligible to download their data. Data collection took place from March 2018 to December 2018, and analysis took place from February 2019 to May 2019. We produced descriptive statistics and pooled models to characterize the level and trajectory of patient uptake over time, reporting linear regression coefficients, 95% CIs, and *P* values. Statistical significance was set at .05, and all tests were 2-tailed. We used Stata version 15.0 (StataCorp) for all results.

The University of California San Francisco institutional review board determined that our project was not human subjects research. This report follows the Strengthening the Reporting of Observational Studies in Epidemiology (STROBE) reporting guideline.

## Results

Of the 12 systems, 4 (33%) were located in the West, 4 (33%) in the Northeast, 3 (25%) in the Southeast, and 1 (8%) in the Midwest. The health systems had a mean annual net patient revenue of $3.64 billion (SD, $1.85 billion; range, $735.79 million to $7.55 billion), mean annual total acute days of 405 878 (SD, 251 667; range, 73 439-997 829), and a mean of 1728 (SD, 956; range, 370-3429) staffed beds.

**Figure 1.**
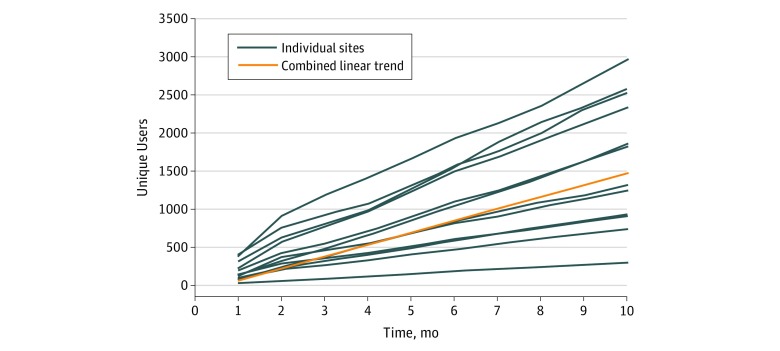
Cumulative New Users of Patient-Facing Application Programming Interface Across 12 US Health Systems

On a month-over-month basis, the rate of unique new users was nearly flat (coefficient, −3.97; 95% CI, −9.82 to 1.88; *P* = .18). On a cumulative basis (ie, adding unique new users to the pool of existing users), this represented a statistically significant increasing linear trend of 156 unique new users per month per health system (95% CI, 123.09 to 188.54; *P* < .001) (Figure 1).

**Figure 2.**
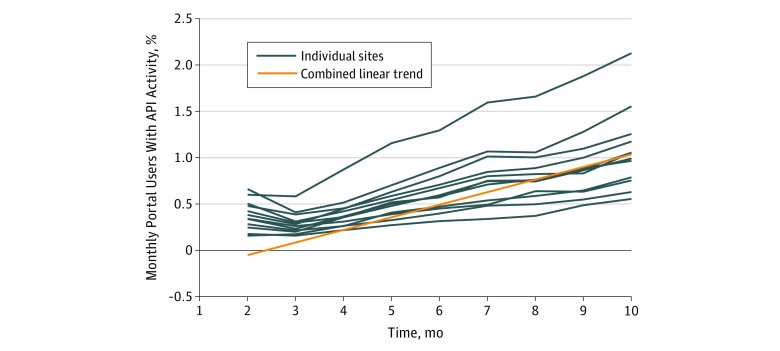
Monthly Users of Patient-Facing Application Programming Interface (API) as a Proportion of Patient Portal Users per Month Across 12 US Health Systems

A mean of 0.7% (SD, 0.4%; range, 0.2%-2.1%) of users who logged into their health care organization’s patient portal in a given month were also users of the API. Over time, there was a statistically significant increasing linear trend in this measure of 0.14% per month per health system (95% CI, 0.10%-0.17%; *P* < .001) (Figure 2).

## Discussion

There are substantial federal policy efforts under the 21st Century Cures Act^1^ and private sector initiatives to advance solutions that allow patients to interact with their health data by first downloading their health record data to their smartphones. To our knowledge, our results offer the first measures of uptake among early adopting health systems and their early adopting patients.

It is anticipated that access to clinical data via Fast Healthcare Interoperability Resources APIs and use of this data by smartphone applications will allow individuals to better understand and control their health data, more easily ensure data accuracy, shop for high-value health care services, avoid the need to repeatedly supply data for entry into each new health care provider’s electronic health record, and increase their participation in clinical research. However, because this capability is new, few applications are currently able to access and use the data. In addition, there has been little effort by health care systems or health information technology vendors to market this new capability to patients, and there are not clear incentives for patients to adopt it.

The generalizability of our findings may be limited because we only included early adopters using an electronic health record from a single vendor; however, more than half of the US population is now served by a health system using this vendor.^3^ Nonetheless, our results offer baseline national measures against which to track efforts to create an ecosystem in which patients use their smartphones to manage and engage with their health record data.
